# A Plea for the London Hospital

**Published:** 1888-04-28

**Authors:** J. F. Kitto

**Affiliations:** Rector of St. Martin's-in-the-Fields.


### A Plea for the London Hospital.

By the Rev. J. F. Kitto, Rector of St. Martin's-in-the-Fields.

I have lived for twenty years as a clergyman amongst the poor of the East-end of London, and therefore may be supposed to know, in some degree at least, the amount of benefit which has been conferred upon the poor by this great hospital which is in their midst. During a considerable portion of that time I was a rector of the parish in which the hospital was situated, and for a still longer period I have taken an active part in the management of its affairs on the House Committee. Therefore I am well qualified to speak with regard to the benefit conferred by this hospital on the poor of the East-end. But it is impossible by any language or any figures to give an adequate impression of the amount of benefit conferred. I do not know where to begin, or what figures to choose to show the advantages. But before I speak on that, I will point out the incidental advantages which seem to arise from the presence of this great hospital. The London Hospital is the greatest of all the metropolitan hospitals, and with it there is connected a medical college. Now, from the medical college the hospital derives a large amount of benefit, because it would be absolutely impossible for the patients to be properly treated if the medical college were not side by side working with it. That we all recognise who have to do with the working of the institution. Then the other advantage I want to point out is, that it is in the hospital, and, as I believe, in the hospital only, that the medical men of the future are at this present moment acquiring the skill which they will by-and-bye exercise for the benefit of the whole community. To whom do we go when we are suffering from ill-health or disease? If any of those in the higher social circles are afflicted with a painful disease, whom do they consult? Take the example of a most illustrious Sovereign, upon whom, at this moment, all our sympathies are turned; his eminent medical attendant was an old student in the London Hospital. And are we not proud of a very distinguished physician of the London Hospital? I do not know whether he actually began his medical career there, but it has culminated in the last few days by his election to the Presidency of the Royal College of Physicians—I allude to Sir Andrew Clark. If he were asked, I am quite confident, from what we know of him, that he would be the first to bear his willing testimony to the fact that it was from the opportunities afforded by the London Hospital (with the committee of which he has worked so faithfully for a number of years) that he was enabled to attain the skill and power which he possesses, and which is at the command of the whole community. I say the cause of the hospital is not simply that of its locality — it is the cause of the highest, the noblest, and the wealthiest of the land. The amount which they contribute is not only an act of philanthropy to their fellows, but it is an assurance to their fellows that when they fall into disease they will have the advantage of medical men, of proper and competent skill to attend them. Referring to the Nursing School—I do not want to take up space by going into detail, but the fact is that the trained nurses of this hospital go out to the whole community. Their skill is acquired in the hospital. The patience, the forbearance, and the skill which they acquire in this hospital are at the service of the whole community at other times.

I think on these grounds there is a great debt due from the community at large, and a duty devolving upon them to subscribe voluntarily to the support of these institutions—institutions which are not supported by the State, and which I for one hope will not be supported by the State. I believe at present, and I hope it will be so for many years to come, that the Christianity and philanthropy of this nation are all-sufficient to induce people to provide funds for the support of this hospital, which is doing such great service. A point to which special attention has been directed is not the benefit which is conferred upon ourselves by the presence of the hospital amongst us, but the benefit which is conferred upon the poor inhabitants. Now, I am aware that in some minds there is a prejudice to some extent against the hospital, on the grounds that the poor are subjected to experiments for the benefit of the rich. I believe that is the greatest caricature of London hospitals. Not only is all the skill which the physicians and surgeons possess, not only is all the attention and care which the nurses give, but all the money that is required for mechanical appliances, and everything in the shape of food and management, are used for the benefit of the patients. There is nothing spared that can possibly be of benefit to a patient: whatever he requires is at once supplied. It does not matter whether he is the poorest patient in the land, when he is once within the walls of the hospital he is regarded with the most constant care. Why, I have known medical men come from the West-end to the East-end of London in the middle of the night, and at all seasons of the year, to see a particular patient who has excited their special attention. There is nothing like the skill and care at our command in our own homes which are at the command of the very poorest patient in the London Hospital. The more intricate and subtle the case may be, the more determined is every one to put forward everything that skill, and science, and care can do to battle with the disease. I say this of itself is an incalculable benefit to be conferred, even if it were only conferred upon a few out of the isolated cases in the East-end of London; but when we become conscious of the fact that it is not conferred merely upon a few isolated cases, but that the whole East-end looks to the London Hospital for the care of its sick poor, then I say the argument is irresistible, and nobody can dispute that the London Hospital is conferring benefit upon the poor which cannot be computed. There were as many as 8,260 in-patients in 1887. I do not know whether people at all realise what that means. Let us try to realise it for a single moment. Take each individual case by itself. A man is being treated at his home. Picture the surroundings of the poor patient's home, in which not only are rest and quiet impossible, not only is proper nursing impossible, not only is proper treatment impossible, but in which it is almost hopeless that the greatest medical skill can baffle the disease. Take that man, and put him in a position which I will not say in every case is certain to cure his disease, but in which in every case he is certain to be provided with every means that we possess towards the cure of his disease. Multiply that case by 8,200, then you have before you the work of the London Hospital. I say that if you individualise these cases, and take them one by one, and present them to the British public, there is not a man or woman who could say "It is no concern of mine whether there is such a hospital or not." There is not one amongst us who has not again and again rejoiced at being able to contribute to the skill and care shown by the hospital staff. I may mention this fact, which is one that affords an argument in favour of these great institutions, and pre-eminently in favour of the London Hospital. It is a hospital which has relieved me in thousands of instances during the time I have been engaged in the East-end of London from a great deal of difficulty. There is a great anxiety which I should have felt in not being able to know what to do for a sick man or a sick woman. But I have felt perfectly relieved and perfectly confident as soon as I had transferred them to the care of the London Hospital.

I would ask what is £30,000 a year after all to this great metropolis? I know that it is very easy to spend, but it is very difficult to get. I do not know whether my computation is quite accurate, but I have counted the number of governors of this hospital. I have found there are are 3,000. We not only want those 3,000, but we want them trebled; at all events, there is not the slightest reason why they should not be doubled, and they would be, if the public knew what the plea is which is put forward. The plea which is put forth is for the sustenance of a hospital which is carrying on a most important work in this part of London. It is an occasion which appeals to our common humanity. I quite recognise the truth that philanthropic efforts are the outcome of religious feeling; but they are not confined to religious feeling; they rest upon a broader and deeper basis than the deepest religious feeling. I rejoice that this cause rests on our common humanity, and whatever may be our religious views, or whether we have no religious views at all, still the voice of our common humanity cries to us from the ground, and we are bound as men, and even more as Christians and religious men; but whether we are Christians, or religious or not, we are bound to support such a cause as this, and one which appeals to us all as this does.

